# Impact of Perfluoropentane Microdroplets Diameter and Concentration on Acoustic Droplet Vaporization Transition Efficiency and Oxygen Scavenging

**DOI:** 10.3390/pharmaceutics14112392

**Published:** 2022-11-05

**Authors:** Rachel P. Benton, Nour Al Rifai, Kateryna Stone, Abigail Clark, Bin Zhang, Kevin J. Haworth

**Affiliations:** 1 Department of Internal Medicine, Division of Cardiovascular Health and Disease, University of Cincinnati, Cincinnati, OH 45267, USA; 2 Cincinnati Children’s Hospital Medical Center, Cincinnati, OH 45229, USA; 3 Department of Pediatrics, University of Cincinnati, Cincinnati, OH 45229, USA

**Keywords:** perfluoropentane microdroplets, cavitation, microfluidic emulsion manufacturing, polydispersity, ultrasound contrast agent, ultrasound duty cycle, intravascular ultrasound

## Abstract

Acoustic droplet vaporization is the ultrasound-mediated phase change of liquid droplets into gas microbubbles. Following the phase change, oxygen diffuses from the surrounding fluid into the microbubble. An in vitro model was used to study the effects of droplet diameter, the presence of an ultrasound contrast agent, ultrasound duty cycle, and droplet concentration on the magnitude of oxygen scavenging in oxygenated deionized water. Perfluoropentane droplets were manufactured through a microfluidic approach at nominal diameters of 1, 3, 5, 7, 9, and 12 µm and studied at concentrations varying from 5.1 × 10^−5^ to 6.3 × 10^−3^ mL/mL. Droplets were exposed to an ultrasound transduced by an EkoSonic^TM^ catheter (2.35 MHz, 47 W, and duty cycles of 1.70%, 2.34%, or 3.79%). Oxygen scavenging and the total volume of perfluoropentane that phase-transitioned increased with droplet concentration. The ADV transition efficiency decreased with increasing droplet concentration. The increasing duty cycle resulted in statistically significant increases in oxygen scavenging for 1, 3, 5, and 7 µm droplets, although the increase was smaller than when the droplet diameter or concentration were increased. Under the ultrasound conditions tested, droplet diameter and concentration had the greatest impact on the amount of ADV and subsequent oxygen scavenging occurred, which should be considered when using ADV-mediated oxygen scavenging in therapeutic ultrasounds.

## 1. Introduction

The application of ultrasound to convert liquid perfluorocarbon droplets into gas microbubbles is known as acoustic droplet vaporization (ADV). ADV is being actively investigated for therapeutic applications, such as targeted drug delivery [1,2,3,4,5,6], thermal ablation [7,8], mechanical ablation [9,10,11], tissue regeneration with acoustically responsive scaffolds (ARS) [12,13,14], and reperfusion injury [15,16]. The later approach relies on gas scavenging that occurs following ADV [17,18,19,20]. The effectiveness of most ADV-based therapies is dependent on the fraction of perfluorocarbon droplets that undergo the phase transition to gas microbubbles, known as the ADV transition efficiency [20].

Fabiilli et al. demonstrated that the ADV transition efficiency improved with increasing droplet diameter when using double-phase perfluorocarbon droplets [21]. These findings were subsequently confirmed by Mercado et al. with the use of relatively polydisperse single-phase perfluorocarbon droplets [22]. It has also been shown that the threshold pressure amplitude necessary to nucleate ADV decreases with the increasing droplet diameter [18,21,22,23].

The relationship between ADV transition efficiency and droplet concentration does not have a single clear trend in the literature. Aliabouzar et al. demonstrated that the ADV transition efficiency was higher for larger droplet volume fractions near the ADV threshold at 2.25 MHz; however, well above the ADV threshold, 1% (*v*/*v*) droplet fractions had a saturating efficiency, whereas 0.05% (*v*/*v*) droplet fractions did not [13]. The authors postulated that acoustic shadowing may cause the reduced ADV transition efficiency at higher concentrations [13]. Dong et al. demonstrated that as the volume fraction of droplets increased, so did the release of the growth factor from double droplets, suggesting a higher ADV transition efficiency with increased droplet concentration [12]. Furthermore, Kang et al. observed that as the droplet concentration increased, the final ADV microbubble diameter decreased, implying a difference in ingassing [19].

The presence of microbubbles in proximity to droplets can impact ADV. Lo et al. found that the ADV threshold could be reduced by co-administering droplets with the ultrasound contrast agent Definity^®^ [24]. The use of microbubbles coupled to microdroplets to nucleate ADV is key to acoustic cluster therapy (ACT), which is being investigated for its potential in ultrasound-mediated drug delivery [1,2,3,25,26]. Studies involving ACT have found that the presence of these clusters enhanced drug delivery and uptake in tumors [2,3]. The increased drug delivery is suggestive of higher ADV transition efficiency as the drug is carried in the microdroplet portion of the cluster and must be released.

Ultrasound parameters can also impact the number of ADV microbubbles formed. Fabiilli et al. observed that as a fluid was exposed to ultrasound multiple times, the ADV transition efficiency increased [21]. The conditions under which this happens can be complex. Kang et al. observed that as the pulse duration or pulse repetition frequency were increased, fewer microbubbles were observed, which they attributed to ADV occurring and the microbubbles subsequently being destroyed by additional ultrasound insonation [19]. This observation is consistent with the fact that they also observed that when fewer microbubbles were present, each microbubble scavenged a larger number of moles of dissolved gas, resulting in larger ADV microbubbles.

To further investigate how the presence of a contrast agent, droplet size, droplet concentration, and ultrasound duty cycle impact ADV, several studies are reported herein. For each of the aforementioned parameters, the ADV transition efficiency was measured by comparing the volume-weighted concentration of perfluoropentane droplets without and with exposure to ultrasound. Additionally, the magnitude of oxygen scavenged was measured in the flow phantom before and during the ultrasound exposure of the fluid containing perfluoropentane droplets. Per our prior work, the magnitude of oxygen scavenged depends on the total volume of perfluoropentane that undergoes ADV per volume of total fluid [20]. As noted above, the impact of droplet diameter and concentration on ADV transition efficiency was investigated in prior studies. In this work, droplets with monodisperse size distributions and modal diameters between 1 and 12 µm were used to minimize the impact of droplet–droplet interactions for droplets of different diameters and to provide additional information that could guide future therapeutic applications of ADV. Similarly, the data reported herein on the effect of the presence of an ultrasound contrast agent are carried out with relatively monodisperse droplet distributions, whereas prior studies used polydisperse droplet distributions [24,25,26].

## 2. Materials and Methods

### 2.1. Droplet Preparation and Characterization 

Kolliphor^®^ P188-coated perfluoropentane droplets of 1, 3, 5, 7, 9, and 12 µm nominal diameters were manufactured with an in-house microfluidic system using a flow-focusing chip with either 5 or 14 µm junctions (model numbers: 300152 and 3200146, Dolomite-Blacktrace, Royston, UK) [27,28]. The microfluidic system was placed at room temperature (23.5 ± 1.5 °C), which is lower than the boiling point temperature of PFP. Table 1 details the manufacturing parameters for the droplets. Kolliphor^®^ P188 was purchased from Sigma Aldrich (St. Louis, MO, USA) and perfluoropentane was purchased from FluoroMed, L.P. (Round Rock, TX, USA). Kolliphor^®^ P188 was dissolved in filtered deionized water from a Nanopure system (model number: 04751, Barnstead, Dubuque, IA, USA). Red food dye (Kroger, Inc., Cincinnati, OH, USA) was used for visualizing fluids during manufacturing. The kinematic viscosity of each polymer with dye solution was measured 5 times using a Cannon-Fenske routine viscometer (Cannon Instrument Company, State College, PA, USA). Droplets were collected in a 50 mL conical vial.

### 2.2. Droplet Size and Stability Measurements 

The modal diameter and concentration of the droplets were measured using a Coulter Counter (Multisizer 4, Beckman Coulter Inc., Brea, CA, USA) equipped with a 30 µm aperture. The system measured droplets between 0.6 to 18 µm in diameter. To reduce the amount of noise in the measurements originating from particulate in the flow phantom, only a subset of diameters was used when calculating the pre and peri ADV droplet concentrations in the effluent. Data were analyzed in bins from 0.6 to 3 µm, 0.6 to 4 µm, 3 to 7 µm, 5 to 9 µm, 7 to 11 µm, and 11 to 13 µm for samples containing 1, 3, 5, 7, 9, and 12 µm, respectively. The measurements were performed by diluting 6 µL of stock solution into 10 mL of phosphate-buffered saline (PBS) for droplets of nominal diameters 3, 5, 7, and 9 µm and 3 µL of stock solution in 10 mL of PBS for droplets of nominal diameters 1 and 12 µm. Volume-weighted size distributions were used to calculate the droplet modal diameter and concentration (mL/mL). The size distribution was used to compute the polydispersity index (PDI, the number-weighted droplet size distribution standard deviation divided by the mean volume-weighted particle diameter, quantity squared) for each droplet. After the measurement, droplets were stored in a freezer at −20 °C, given their instability observed in pilot experiments at 4 °C, and used within 45 days.

Due to Ostwald ripening [29,30] and potential spontaneous droplet vaporization during storage, a time-dependent stability study of the 1, 3, 5, 7, 9, and 12 µm nominal diameter droplets was conducted. On the day of manufacturing (D0), droplets from a stock solution were aliquoted into multiple Eppendorf tubes and stored at −20 °C. For each measurement, droplets in one Eppendorf tube were thawed and the size distribution was measured every day for the first week and every four days up to day 33.

### 2.3. In Vitro Experimental Setup

**Figure 1:**
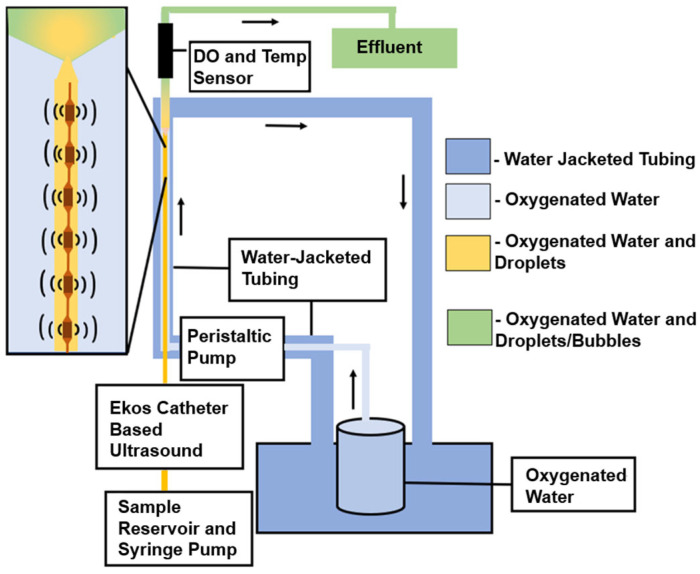
Schematic of the in vitro flow phantom with black arrows showing direction of flow. Droplets were pumped from the sample reservoir and through the flow system maintained at 37 °C. The oxygenated water was saturated to a PO_2_ of 560.8 ± 7.6 mmHg. Droplets were exposed to a 2.35 MHz-pulsed ultrasound using a 6 cm treatment zone EkoSonic^®^ catheter. The PO_2_ in the fluid distal to the catheter was measured by a flow-through oxygen sensor. The effluent was collected to measure the size distribution and volume concentration of droplets that did not undergo ADV.

An in vitro flow phantom was used to perform experiments testing the ADV transition efficiency and oxygen scavenging (Figure 1). The flow phantom was constructed using 3.18 mm of inner diameter EVA tubing (McMaster-Carr, Aurora, OH, USA). The tubing was water-jacketed, such that all ADV was performed at a temperature of 37 ± 1 °C, which was monitored by an internal thermocouple immediately upstream to the site of the oxygen partial pressure (PO_2_) measurement. A peristaltic pump was used to induce 16 mL/min flow of oxygenated deionized water (Section 2.1) through the flow phantom. The oxygenated deionized water was prepared in a covered 4 L reservoir by constantly bubbling a 95%/5% O_2_/CO_2_ gas mixture through it as is typically used for ex vivo systems. The oxygenated water’s temperature was maintained in the range of 36.5 to 38.0 °C with a water bath [31]. The corresponding PO_2_ of the oxygenated water was 560.8 ± 7.6 mmHg.

An EkoSonic^®^ catheter (Boston Scientific, Model 500-56112, Natick, MA, USA) was inserted into the flow phantom using a hemostasis valve. The EkoSonic^®^ catheter is composed of a MicroSonic™ Device (MSD) that contained 6 pairs of ultrasound transducer elements (used to nucleate ADV) inserted into an Intelligent™ Drug Delivery Catheter (IDDC). The EkoSonic^®^ catheter is designed to be disposable in the clinical environment. However, due to cost, the catheters were reused over multiple experiments, with the catheters flushed with deionized water between trials. The health of the MSD was assessed each day via an impedance measurement (Aim4170D, Array Solutions, Sunnyvale, TX, USA). Based on pilot data, MSDs were only used if the phase of the impedance at 2.35 MHz (the ultrasound insonation frequency) was between –15 and 10 degrees. Droplets were diluted (5 × 10^−4^ mL/mL, unless noted otherwise) in deionized water taken from the reservoir and infused through the coolant port of the IDDC via a syringe pump operating at 5 mL/min. This flow rate matched prior ADV studies [16]. A 1-inch stir bar was placed within the syringe and manually agitated to prevent droplets from settling. Unless as noted below, the EkoSonic^®^ catheter was driven with a 40-cycle tone burst with a burst period of 1.000 ms (AFG3500B, Keysight Technologies, Inc., Santa Rosa, CA, USA) through a power amplifier (2200L, Electronics and Innovation, Ltd., Rochester, NY, USA). A 23B RF Wattmeter (Sonic Concepts, Inc., Bothell, WA, USA) was used to monitor the electrical drive power to the catheter and the applied voltage was adjusted to ensure a pulse-average power of 47 W, the maximum output power used in FDA-cleared protocols. The acoustic field pattern from a transducer pair in the catheter is complex [32].

The droplet stock concentration was measured and appropriately diluted to the target concentration for the experiment. The droplets were then infused through the coolant port and into the oxygenated water. The PO_2_ was measured downstream using an inline dissolved oxygen and temperature flow-through sensor (TOFTC2, PyroScience GmbH, Aachen, Germany). The flow time from the tip of the EkoSonic^®^ catheter to the flow-through sensor was 3 s. Measurements were made over 40 s periods with the droplet syringe pump off (pre-droplets), droplet syringe pump on but without driving the EkoSonic^®^ catheter (peri-droplets), and droplet syringe pump on with the EkoSonic^®^ catheter driven at 47 W (peri-ADV). All experiments measuring the PO_2_ and ADV transition efficiency were repeated five times. The size distribution of droplets in the effluent was measured (Section 2.2). Care was taken to remove any bubbles that floated to the top of the effluent. The ADV transition efficiency was calculated by taking one minus the ratio of the total volume concentration of the peri-ADV effluent to the peri-droplet effluent.

### 2.4. Effect of Lumason on ADV-Mediated Oxygen Scavenging

The effect of the ultrasound contrast agent Lumason^®^ (Bracco Diagnostics, Inc., Milan, Italy) on the ADV transition efficiency and oxygen scavenging was studied by comparing measurements with and without Lumason^®^ co-administered with droplets. Lumason^®^ was activated according to the manufacturer’s package insert and used within 48 h of activation. A 19 G needle (Hamilton, Reno, NV, USA) connected to a 25 µL gas-tight syringe (Becton Dickinson, Franklin Lakes, NJ, USA) was used to withdraw 24 µL of Lumason^®^. Lumason^®^ was transferred to a 60 mL syringe (Becton Dickinson, Franklin Lakes, NJ, USA) containing 60 mL of 95% DI water and perfluorocarbon droplets (4.8 × 10^−4^ ± 0.6 × 10^−4^ mL/mL final concentration). The Lumason^®^ dose was based on the package insert dose per weight (0.03 mL/kg). Co-diluted Lumason^®^ and droplets were infused through the coolant delivery port of an EkoSonic^®^ catheter with a Harvard Apparatus Elite syringe pump (Harvard Apparatus, Holliston, MA, USA) and exposed to ultrasound, as described in Section 2.3.

### 2.5. Effect of Ultrasound Duty Cycle on ADV-Mediated Oxygen Scavenging

The effect of ultrasound duty cycle was determined by fixing the pulse duration at 40 cycles (17.0 µs) and varying the burst period (0.450 ms, 0.725 ms, and 1.000 ms) or by fixing the burst period at 1.000 ms and varying the pulse duration (17.0 µs, 23.4 µs, and 37.9 µs). These combinations were selected so that varying the burst period or the pulse duration corresponded to the duty cycles of 1.70%, 2.34%, or 3.79%. The time-average electrical drive power used in these studies was near the maximum operating range for the single-use MSD, and thus, higher duty cycles could not be tested without damaging the catheter. Because the transition efficiency and oxygen scavenging did not change substantially between droplets of nominal diameters of 7, 9, and 12 µm, the effect of ultrasound duty cycle was only investigated for droplets with nominal diameters between 1 and 7 µm. The initial volume-weighted droplet concentrations were 5.0 × 10^−4^ ± 0.4 × 10^−4^ mL/mL and 4.8 × 10^−4^ ± 0.6 × 10^−4^ mL/mL when investigating the effect of burst period and pulse duration, respectively.

### 2.6. Effect of Droplet Concentration on ADV-Mediated Oxygen Scavenging

The effect of droplet concentration on the ADV transition efficiency and oxygen scavenging was measured for droplets with nominal diameters of 3, 5, and 7 µm. Droplets of nominal diameter 9 and 12 µm were not investigated as described in Section 2.5. The 1 µm nominal diameter droplets were not investigated because the droplet production rates were too slow. The volume-weighted droplet concentrations used were between 5.1 × 10^−5^ and 6.5 × 10^−3^ mL/mL.

### 2.7. Comparison of Measured Oxygen Scavenging to the Transition Efficiency-Based Model

For each of the experiments described in Section 2.4, Section 2.5 and Section 2.6, a previously reported physics-based model was used to estimate the magnitude of oxygen scavenging based on the volume of perfluoropentane transitioned from a liquid to a gas (i.e., the transition efficiency times the initial volume-weighted droplet concentration) [20]. Within the model, the relative partial pressures in the peri-droplet fluids were 166 mmHg, 560 mmHg, 26.7 mmHg, 2.0 mmHg for N_2_, O_2_, CO_2_, and Ar, respectively. The vapor pressure of water was 47 mmHg. These values were based on the measured PO_2_ values and then assuming the remaining partial pressures of gases were proportionate to their composition in standard air. An ambient hydrostatic pressure of 1 atm and a temperature of 37 °C were assumed. Perfluoropentane was assumed to be insoluble in all fluids and the solubility of oxygen was assumed to be 964 L atm/mol in water [33,34]. The expansion factor was 125, based on the change in density of liquid and gaseous perfluoropentane [20]. Surface tension and viscosity were not taken into account for the numerical model.

### 2.8. Statistical Analysis

The amount of oxygen scavenging and transition efficiency for the absence and presence of Lumason^®^ was compared using a 2-tailed Mann–Whitney analysis. A Kruskal–Wallis analysis for each droplet diameter with Dunn’s multiple testing correction was used to compare the amount of oxygen scavenging and transition efficiency for studies of the ultrasound duty cycle. The agreement between the measured and modeled oxygen scavenging was evaluated using an intraclass correlation coefficient with 95% confidence intervals and the correlation was assessed using Pearson correlation coefficients. We selected the consistency and a two-way mixed-effects ICC model of a single score per observed measurement type (C,1) [35]. The scores were defined as measurements predicted by the numerical oxygen scavenging model. All statistical analyses were performed using Prism 9 (GraphPad Software Inc., La Jolla, CA, USA) and SAS, Version 9.4 (SAS Institute, Cary, NC, USA). A *p*-value of less than 0.05 was used to determine the significant differences for all statistical tests.

## 3. Results

### 3.1. Droplet Manufacturing and Characterization

**Figure 2:**
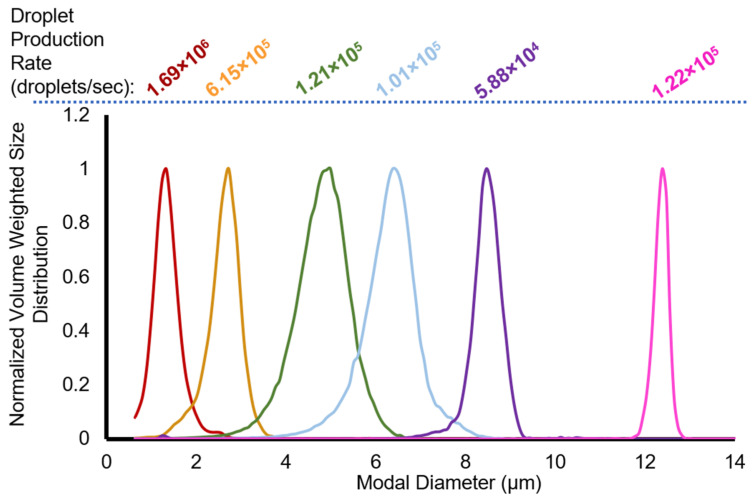
Normalized volume-weighted distribution for microfluidic droplets of nominal diameters 1, 3, 5, 7, 9, and 12 µm. The droplet production rate (droplets/s) is listed at the top of the figure for each droplet diameter. The colors (red, yellow, green, blue, violet, and magenta) used to plot the data correspond to nominal droplet diameters for each microdroplet (1, 3, 5, 7, 9, and 12 µm, respectively) and are used for all subsequent figures.

In general, polymer solution viscosity increased with polymer concentration (Table 1). The normalized volume-weighted size distribution of the droplets is shown in Figure 2. As the microfluidic chip junction decreased from 14 µm to 5 µm, the droplet size decreased. Generally, for both chips used, as the ratio of polymer flow rate to the perfluoropentane flow rate increased, the modal diameter decreased. However, this trend was not observed with the 9 µm droplets. The calculated droplet production rate (volume of PFP per droplet divided by the PFP flow rate) agreed within a factor of two with the measured droplet production rate (Table 1).

### 3.2. Droplet Stability at −20 °C

Table 2 lists the nominal diameter, modal diameter, and polydispersity index (PDI) at day 0 (D_0_) and at day 33 (D_33_). The modal diameter remained constant over 33 days, except for the 5 µm nominal diameter droplets, which size-shifted 80 nm when stored at −20 °C. The PDI increased during storage primarily due to an increase in measured particulate less than 1 µm in diameter. The average PDI for each nominal droplet size across all time points measured was 0.102 ± 0.0216, 0.165 ± 0.0561, 0.2292 ± 0.13, 0.285 ± 0.137, 0.536 ±0.003, and 0.09 ± 0.068 for 1, 3, 5, 7, 9, and 12 µm, respectively. The concentration stability was measured for 7 µm droplets and varied by less than 6% over 33 days. The modal diameter of droplets measured before and after use within the in vitro system (~3 min at 37 °C) showed no change.

### 3.3. Effect of Lumason^®^ on ADV-Mediated Oxygen Scavenging

**Figure 3:**
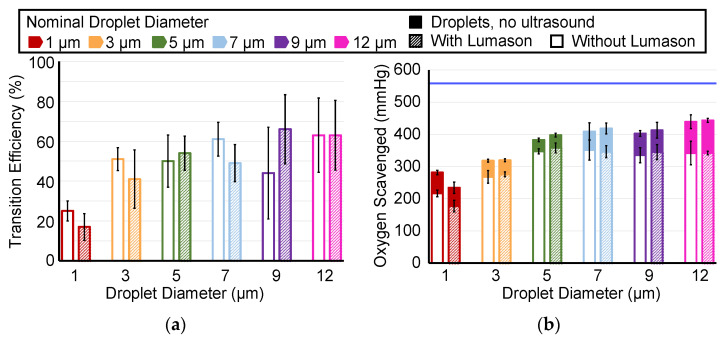
(**a**) The measured ADV transition efficiency without (white fill) and with (hatched fill) Lumason^®^ as a percentage of the change in the volume-weighted size distribution of the droplets with and without ADV. (**b**) The amount of oxygen scavenging with and without Lumason^®^. The solid filled top of the stacked bar graph is the amount of oxygen scavenging observed peri-droplet (i.e., no ultrasound exposure, and thus, no ADV). The bottom portion of each bar describes the amount of oxygen scavenging observed peri-ADV without (white fill) and with (hatched fill) Lumason^®^. The horizontal blue bar represents the initial PO_2_ before droplets were infused, and thus, the maximum amount of PO_2_ that could be scavenged. The error bars denote the standard deviation for five samples. The legend applies to both panels.

ADV transition efficiency and oxygen scavenging were compared each with and without Lumason^®^ for droplet diameters between 1 and 12 µm (Figure 3). The total oxygen scavenging was composed of the effect of the addition of droplets without ultrasound (i.e., peri-droplet and pre-ADV, solid color Figure 3b) and the effect of ADV (peri-ADV, white or hatched component Figure 3b). Oxygen scavenging due to droplets without ADV was expected due to the solubility of oxygen in liquid perfluoropentane [20]. Experiments performed without droplets measured no statistically significant change in the oxygen partial pressure with and without Lumason (−0.2 ± 1.7 mmHg). Both ADV transition efficiency and oxygen scavenging had an increasing trend with increasing droplet diameter between 1 and 7 µm, with minimal differences between 7, 9, and 12 µm droplets. The magnitude of the difference between oxygen scavenging with or without Lumason^®^ was relatively small compared to the total amount of oxygen scavenged. The average difference between the amount of oxygen scavenging with and without Lumason^®^ across all droplet diameters was 2.67 ± 19.03 mmHg. The addition of Lumason^®^ resulted in increased oxygen scavenging when using 5 µm droplets (14.85 mmHg, *p*-value < 0.05) and decreased oxygen scavenging with 1 µm droplets (32.3 mmHg, *p*-value < 0.05). The transition efficiency with and without Lumason^®^ was different only for droplets of nominal diameter 7 µm (12 percentage points, *p*-value < 0.05). There was significantly less (*p*-value < 0.05) oxygen scavenging observed with 1 µm droplets than with 7 µm, 9 µm, and 12 µm droplets when Lumason^®^ was added (15 comparisons). Additionally, the ADV of 3 µm droplets with Lumason^®^ resulted in less oxygen scavenging than using 12 µm droplets. The same pairs of significant differences in oxygen scavenging between droplet diameters without the addition of Lumason^®^ were the same as with Lumason^®^, except no difference was observed between 1 and 9 µm droplets. The transition efficiency was only different with 12 µm and 9 µm droplets when compared to 1 µm droplets. Without Lumason^®^, the transition efficiency was only different with 7 µm and 12 µm droplets in comparison to 1 µm droplets.

### 3.4. Effect of Ultrasound Duty Cycle on ADV-Mediated Oxygen Scavenging

**Figure 4:**
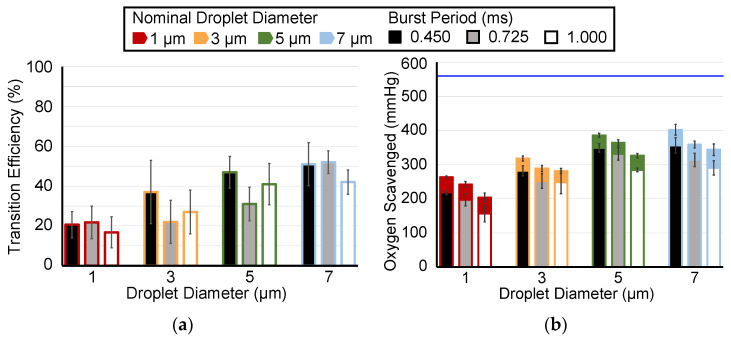
(**a**) ADV transition efficiency with burst periods of 0.450 ms (3.79% duty cycle), 0.725 ms (2.34% duty cycle), and 1.000 ms (1.70% duty cycle). The transition efficiency is calculated as a percent change based on the volume-weighted size distributions of the droplets. (**b**) The measured amount of oxygen scavenging peri-droplet (colored fill) or peri-ADV with burst periods of 0.450 ms, 0.725 ms, and 1.000 ms for droplets of 1, 3, 5, and 7 µm diameters. The horizontal blue bar represents the initial PO_2_ before droplets were infused, and thus, the maximum amount of oxygen that could be scavenged. The error bars denote the standard deviation for five samples. The legend applies to both panels.

**Figure 5:**
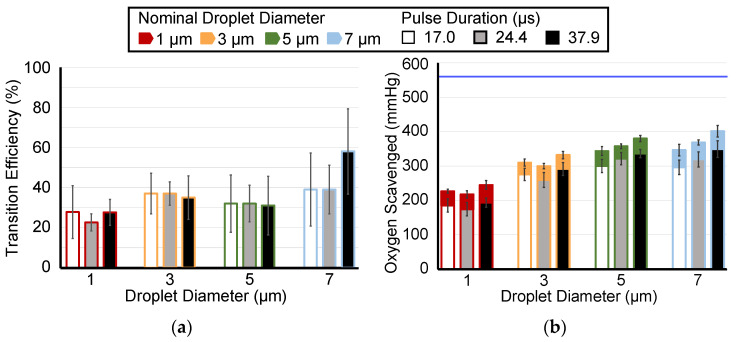
(**a**) ADV transition efficiency with pulse durations of 17.0 µs (1.70% duty cycle), 23.4 µs (2.34% duty cycle), and 37.9 µs (3.79% duty cycle). The transition efficiency is calculated as a percent change based on the volume-weighted size distributions of the droplets. (**b**) The measured amount of oxygen scavenging peri-droplet (colored fill) or peri-ADV with pulse durations of 17.0 µs, 23.4 µs, and 37.9 µs for droplets of 1, 3, 5, and 7 µm diameters. The horizontal blue bar represents the initial PO_2_ before droplets were infused, and thus, the maximum amount of PO_2_ that could be scavenged. The error bars denote the standard deviation for five samples. The legend applies to both panels.

For each nominal droplet diameter, the ADV transition efficiency did not show a consistent trend as a function of burst period (Figure 4a) or pulse duration (Figure 5a). There were no statistically significant differences in ADV transition efficiency for the varying pulse duration. The only statistically significant difference in transition efficiency for the varying burst period was observed between 0.450 ms and 0.725 ms (17 percentage points, *p*-value < 0.05). For all burst periods and all pulse durations (except at 1 µm), a trend existed for increasing oxygen scavenging with increasing duty cycle. More oxygen was scavenged with a 0.450 ms burst period than with a 1.000 ms burst period with 1, 3, 5, and 7 µm droplets (Figure 4a, *p*-value < 0.05). However, there was not a statistically significant difference in oxygen scavenging between 0.750 ms and the other two burst periods. More oxygen was scavenged with a 37.9 µs pulse duration than with 17 µs for 5 and 7 µm droplets, and there was a statistically significant difference between 17.0 µs and 23.4 µs for 3 µm droplets (Figure 5a, *p*-value < 0.05). Regardless of the burst period or pulse duration, there was significantly less oxygen scavenging observed with 1 µm droplets than with either 5 or 7 µm droplets (6 comparisons). Moreover, the transition efficiency was also significantly different between 1 µm and 7 µm droplets for all ultrasound parameters tested except for the 17.0 µs pulse duration. A significant difference in transition efficiency between 3 and 7 µm droplets was also observed for 0.725 ms burst period and 37.9 µs pulse duration; and a difference between 1 and 5 µm for 1.000 ms burst period.

### 3.5. Effect of Droplet Concentration on ADV-Mediated Oxygen Scavenging

**Figure 6:**
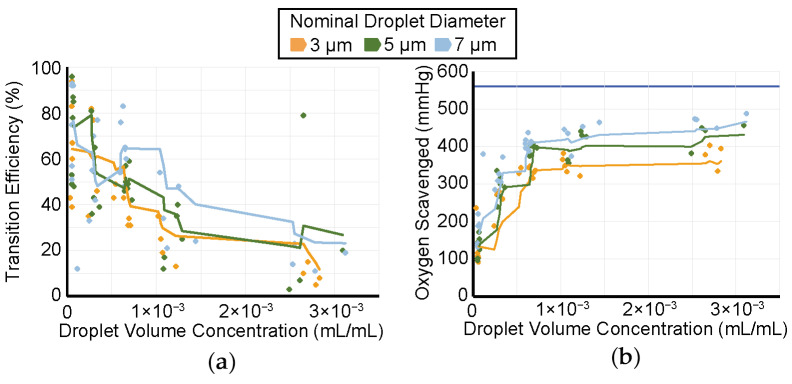
(**a**) ADV transition efficiency for droplets of nominal diameters 3 (yellow), 5 (green), and 7 (blue) µm for initial droplet concentrations between 5.10 × 10^−5^ to 6.30 × 10^−3^ mL/mL without Lumason. The transition efficiency is calculated as a percent change based on the volume-weighted size distributions of the droplets. Individual measurements are denoted with diamonds. Lines are the moving average of the measurements with a period of 6. (**b**) The measured amount of oxygen scavenging for droplets of nominal diameters 3 (blue diamonds), 5 (green diamonds), and 7 (yellow diamonds) µm with corresponding lines representing moving averages. The horizontal blue bar represents the initial PO_2_ before droplets were infused, and thus, the maximum amount of PO_2_ that could be scavenged. The legend applies to both panels.

The ADV transition efficiency and magnitude of oxygen scavenging for droplets with nominal diameters of 3, 5, and 7 µm are shown in Figure 6. A moving average line with a period of 4 was calculated and plotted to facilitate the visualization of the trend that the ADV transition efficiency decreased (Figure 6a) and oxygen scavenging increased (Figure 6b) with the increasing droplet volume-weighted concentration. The largest changes in transition efficiency and oxygen scavenging occurred for volume concentrations up to 0.001 mL/mL. Note that although the ADV transition efficiency decreased (i.e., the percentage of droplets that phase-transitioned) with increasing droplet volume concentration, the total volume of droplets that phase-transitioned was observed to increase, which is consistent with the observations in Figure 6b.

### 3.6. Comparison of Measured Oxygen Scavenging to the Transition Efficiency-Based Model

**Figure 7:**
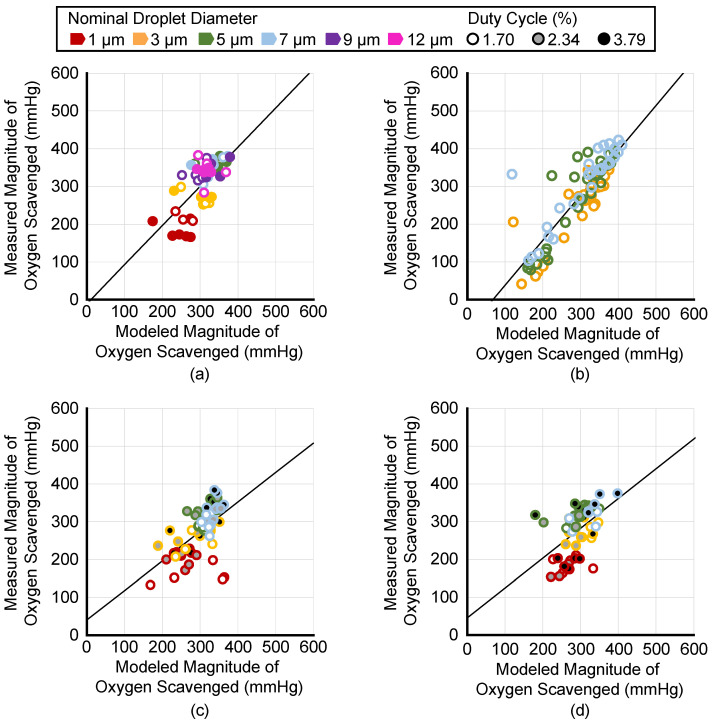
The measured amount of oxygen scavenged versus the modeled amount of oxygen scavenged for (**a**) trials investigating the impact of Lumason^®^, (**b**) droplet concentration, (**c**) burst period, and (**d**) pulse duration. The legend at the top applies to all panels. Data points represent individual trials with the outer marker color indicating the nominal droplet diameter and the fill indicating the duty cycle, except in panel (**a**) where filled data points are from trials that used Lumason^®^ and open data points did not. The solid black line is a best-fit line based on the Pearson correlation analysis.

The ADV-mediated measured oxygen scavenging was compared to the predicted oxygen scavenging based on the Radhakrishnan model that uses measured transition efficiency as an input [20]. Figure 7 plots the measured versus modeled oxygen scavenging values for each trial. For readability, the data are separated by whether the data were used to compare the effect of Lumason^®^ (Figure 7a), droplet concentration (Figure 7b), burst period (Figure 7c), and pulse duration (Figure 7d). The ICC and 95% confidence intervals were 0.62 (0.47, 0.73), 0.55 (0.38, 0.68), 0.45 (0.26, 0.60), and 0.84 (0.78, 0.88) for the Lumason^®^ trials, burst period trials, pulse duration trials, and droplet concentration trials, respectively. Collectively, the ICC and 95% confidence interval for all trials was 0.72 (0.67, 0.76). The Pearson correlation coefficient and 95% confidence intervals were 0.67 (0.50, 0.79), 0.57 (0.37, 0.72), 0.50 (0.28, 0.67), 0.88 (0.82, 0.91), and 0.76 (0.71, 0.81), for Lumason^®^ trials, burst period trials, pulse duration trials, droplet concentration trials, and all trials, respectively.

## 4. Discussion

### 4.1. Droplet Manufacturing and Characterization

The current study showed that the droplet diameter would decrease as the ratio between the polymer surfactant flow rate and PFP flow rate increased. However, it was observed that smaller microfluidic junctions were necessary to achieve the smallest diameter droplets. Although manufacturing droplets of specific diameters was repeatable, the production rates decreased for smaller droplets. Running the chip overnight for 1 µm droplets resulted in sufficient droplet stock to run approximately 4 trials with dilutions of 5 × 10^−4^ mL/mL. These production rates may be limiting in certain applications. Storage stability is an important parameter for low-boiling point PFC-in-water droplets to be of practical use, which was a feature of the droplets in this study. The modal diameter did not change substantially over 33 days. However, the polydispersity increased, primarily due to increased particle counts at diameters of 1 µm and smaller.

### 4.2. Effect of Lumason^®^ on ADV-Mediated Oxygen Scavenging

In the present study, the effect of using the ultrasound contrast agent Lumason^®^ showed no statistically significant effect in most cases, and where it did, the effect was inconsistent (8.5% increase and 2.6% decrease for 1 and 5 µm droplet, respectively). Prior studies have demonstrated that ultrasound contrast agents decrease the ultrasound pressure amplitude necessary to nucleate ADV (nominally 6 MPa to 2 MPa peak rarefactional pressure) using 20 µs pulses at a burst period of 2 ms at 1.44 MHz (1% duty cycle) [24]. In that study, Definity^®^ was used at concentrations of 10^3^, 10^4^, and 10^5^ microsphere per mL with highly polydisperse droplets at a concentration of 5.3 × 10^7^ droplets/mL. Based on the Lumason^®^ package insert concentration (1.5 to 5.6 × 10^8^ microspheres/mL), our studies were performed at a concentration of 8.4 × 10^6^ microspheres/mL in the IDDC coolant port, assuming no loss of Lumason^®^ during infusion. The droplet volume fraction for our study (5 × 10^−4^ mL of droplet volume per mL of diluent) corresponded to droplet concentrations of 9.6 × 10^8^ and 5.5 × 10^5^ droplets/mL for 1 and 12 µm droplets, respectively. Thus, the ultrasound insonation parameters, droplet concentrations, and ultrasound contrast agent concentrations are similar between the studies. Given the differing impact of ultrasound contrast agents between the work of Lo et al. and those reported herein, it may be inferred that, as long as the ultrasound insonation pressure amplitude is greater than the ADV threshold pressures, reducing the threshold may not correspond to increased ADV transition efficiencies. There are notable differences between the work of Lo et al. and the results of the present study that prevent a definitive conclusion. Key differences include the droplet size distributions (polydisperse in Lo et al. versus monodisperse in the present study) and the ultrasound beam profile (relatively uniform in Lo et al. and complex from an EkoSonic^®^ catheter [32]). Additionally, a broader range of ultrasound contrast agent concentrations in our study may have produced more pronounced differences. However, decreasing the ultrasound contrast agent concentration to be more similar to Lo et al. would likely decrease any observed effect. A final key difference between the two studies is that Lo et al. observed the greatest effect of pulse duration when millisecond-long pulses were used. These pulse durations were not possible at the pulse repetition frequencies used in this study without causing damage to the catheters. When studying acoustic cluster therapy, where microbubbles and microdroplets are electrostatically bound, Healey et al. observed that the nucleation efficiency decreased when albumin was added to a solution, which can cause an uncoupling of the microbubbles and microdroplets [25]. Their work suggests that increasing the distances between droplets and microbubbles decreases the impact of the microbubble on transitioning the droplet. Neither the work of Lo et al. nor that reported herein studied whether any microbubble and microdroplets were clustered together.

### 4.3. Ultrasound Insonation Parameters

Both the pulse duration and burst period affect the amount of oxygen scavenging during ADV. The burst period had the greater impact, although the effect was a modest increase in oxygen scavenging (10.8% ± 0.2%). Our observation that increasing the ultrasound duty cycle increased the ADV transition efficiency (and oxygen scavenging) is in concert with the observation from Lo et al. [24] that the ADV threshold pressure decreased with increasing duty cycle. However, as discussed in Section 4.2, there was not a coherence between ADV transition efficiency and ADV threshold pressure amplitude in the presence of an ultrasound contrast agent. Additional studies without ultrasound contrast agents would be needed to further elucidate the relationship between the ADV transition efficiency and threshold pressure amplitude. A limitation of this work is that hardware limitations restricted the range of ultrasound duty cycles from 1.70% to 3.79%.

Varying ultrasound insonation parameters did not substantially affect oxygen scavenging and, for the most part, transition efficiency when the droplet diameter was varied. In agreement with our observations made in experiments with and without the ultrasound contrast agent, using 1 µm and, in part, 3 µm droplets, result in significantly less oxygen scavenged than with 7, 9, or 12 µm droplets when varying duty cycles. However, differences in transition efficiency were not as consistent as those in oxygen scavenging within and between groups.

### 4.4. Effect of Droplet Concentration on Ultrasound Mediated Oxygen Scavenging

The current study observed that as droplet concentration increased, the amount of ultrasound-mediated oxygen scavenging increased. The PO_2_ reduction observed within the surrounding fluid with increasing droplet concentrations showed a saturating effect at around 400 mmHg (approximately 70% of the total PO_2_). Kang et al. observed with increasing droplet concentrations that the ADV microbubbles were smaller [19]. The ADV microbubbles measured by Kang et al. included ingassing, and the smaller microbubble sizes may indicate that at higher concentrations, there is less ingassing per microbubble, which would be consistent with the saturation effect observed in Figure 6 and the theoretical model of oxygen scavenging plotted by Mercado-Shekhar et al. [16]. At concentrations below approximately 5 × 10^−4^ mL/mL, the magnitude of oxygen scavenging has a strong dependence on concentration. For nanodroplets, Yang et al. observed a decrease in ADV threshold pressure amplitude (8.5 MPa to 5.6 MPa peak rarefactional), as determined by changes in B-mode echogenicity, for increasing droplet concentrations (10^4^ to 10^8^ droplets/mL) [36]. Notably, these pressure amplitudes are significantly greater than the pressure amplitudes used in this study (1.5 MPa), though this could be consistent with the decreasing transition efficiency with a decreasing droplet diameter.

The total volume of PFP that phase-transitioned increased with increasing droplet concentrations, consistent with the increased oxygen scavenging (Figure 7). However, even though more oxygen scavenging was observed with increasing droplet concentrations, the transition efficiency as a percent of infused droplets decreased at higher droplet concentrations. The results of this study are not able to indicate the reason for the decrease, but it is possible that acoustic shadowing or droplet–droplet interactions are a reason. Supplemental Table S1 provides the average distance between droplets for each nominal diameter at the lowest and highest droplet concentration in these studies. Aliabouzar et al. observed a complex relationship between droplet concentration and ADV transition efficiency for double emulsion microdroplets. At higher droplet concentrations (1%), the ADV transition efficiency saturated with an increasing insonation pressure amplitude, while lower concentrations (0.05%) showed a linear increase up to 6.5 MPa [13]. Similarly, Dong et al. observed that, at 3.3 MPa insonation, the amount of growth factor released from an ADV double droplet was similar for 0.25% and 1% droplet concentrations [12]. However, at 8.8 MPa insonation, the higher droplet concentration released more growth factors, suggesting a higher ADV transition efficiency [12]. Together, these results highlight that the absolute trends observed in our studies may vary depending on the ultrasound pressure amplitude, which was not studied herein.

### 4.5. Comparison of Measured Oxygen Scavenging to Transition Efficiency-Based Model

Reasonable agreement was observed between the Radhakrishnan analytic model of oxygen scavenging [20], which is based on the measured amounts of PFP that phase-transitioned per milliliter of fluid, and the measured amount of oxygen scavenging droplet concentrations of 5 × 10^−4^ mL/mL (Figure 7a). The Pearson correlation coefficient quantifies that the data in Figure 7b demonstrate a strong linear relationship between the measured and modeled magnitude of oxygen scavenging as the droplet concentration was varied; however, there was an offset in the slope and intercept. This effect is observed with the intraclass correlation coefficient being slightly lower than the Pearson correlation coefficient. Potential reasons for the disagreement between measured and modeled values may include errors in the estimated volume expansion of the PFP, particularly because of surface tension [18,37] or the measured transition efficiency. In all experiments, the coefficient of variance was larger for the measured transition efficiency than the measured oxygen scavenging. Correspondingly, significant differences between experimental groups were more often seen in the magnitude of oxygen scavenging than the transition efficiency due to the larger standard deviation in the transition efficiency measurements (Figure 3, Figure 4 and Figure 5). Fabiilli et al. [21] and Radhakrishnan et al. [20] also observed relatively large transition efficiency standard deviations, similar to this study, though their large standard deviations may be due to the use of polydisperse droplet distributions. Despite potential errors in the model, measuring the oxygen scavenging and reversing the model to compute an estimated transition efficiency measurement could be a more accurate way to obtain transition efficiency values. Additional studies designed to estimate the precision and accuracy of this approach would be needed to validate it. Alternative particle sizing techniques, such as nanoparticle tracking analysis [38] that can measure particles below the limits of a Coulter counter, could provide more accurate and less variable estimates of the transition efficiency. An additional source of error is that the ADV microbubbles are assumed to remain microbubbles. However, depending on the temperature, size, and surface tension, it is possible for droplets to recondense [37,39].

### 4.6. Study Limitations

The large variability observed with transition efficiency for pulse duration, burst period, and Lumason^®^ trials could be attributed to challenges in measuring effluent samples. Our measurements assume that only droplets and not bubbles are measured. When measuring peri-ADV samples, bubbles floating to the top of the sample were removed. Even though care was taken to only remove bubbles, approximately 10–100 µL of the sample was also removed in the peri-ADV samples. Another limitation with the method used to measure samples showed that over time, droplets within a sample will settle to the bottom. Larger droplets settle to the bottom faster than smaller droplets. Although samples were mixed well before each measurement, the droplets would settle during the measurement (note that the terminal velocity is between 4 and 14 mm/s for perfluoropentane droplets between 1 and 12 µm in diameter in water).

A limitation of the current study was the re-use of the single-use EkoSonic^®^ catheters due to their cost. At the beginning of each experimental day, an electrical impedance measurement was taken from the EkoSonic^®^ device. If the phase was between –15 to 10 degrees, the EkoSonic^®^ device was used. In pilot experiments, it was observed that the magnitude of ADV varied outside of this range. When the phase was greater than 10 degrees, the amount of ADV was higher and below −15 degrees it was lower. The pilot experiments demonstrated that the phase of the impedance changed relatively quickly the first several times a previously unused catheter was used. After that, the amount of ADV stabilized and the catheter could be used for approximately 70 to 120 trials. Therefore, the first six trials of a new catheter were not used in the final data analysis.

## 5. Conclusions

The presented results indicate that the ADV transition efficiency and magnitude of oxygen scavenging when infusing perfluoropentane droplets through the coolant port of an EkoSonic^®^ device driven at 47 W does not change substantially in the presence of Lumason^®^ at a concentration based on clinical dosing. A modest effect was observed where more ADV occurs as the ultrasound duty cycle was increased from 1.70% to 3.79%. More significant changes in transition efficiency and oxygen scavenging were observed as the droplet concentration was increased from 5 × 10^−5^ mL/mL to 5 × 10^−4^ mL/mL, but then saturated for higher concentrations. These results indicate the oxygen scavenging effect can be modulated most readily by varying the droplet concentration. Furthermore, alternate approaches to increasing the transition efficiency at higher droplet concentrations may provide a route to overcoming the saturation effect.

## Figures and Tables

**Table 1:** Droplet manufacturing parameters for polymer flow rate, PFP flow rate, and polymer concentration are listed. For droplets of 1 and 3 µm nominal diameters, a chip with a 5 µm junction was used. For droplets of 5, 7, 9, and 12 µm nominal diameters, a chip with a 14 µm junction was used. Measured polymer viscosity, measured production rate, and anticipated production rate (based on nominal droplet diameter and PFP flow rate) are listed.

Nominal Droplet Diameter (µm)	Polymer Flow Rate (µL/min)	PFP Flow Rate (µL/min)	Polymer Concentration (mg/mL)	Polymer Kinematic Viscosity (cp)	Measured Production Rate (droplets/sec)	Anticipated Production Rate (droplets/sec)
1	8.33	0.1	45	2.23 ± 0.02	1.69 × 10^6^	2.53 × 10^6^
3	5	0.3	45	2.23 ± 0.02	6.15 × 10^5^	4.69 × 10^5^
5	11.67	0.8	105	4.79 ± 0.01	1.21 × 10^5^	1.94 × 10^5^
7	11.67	1.2	105	4.79 ± 0.01	1.01 × 10^5^	1.00 × 10^5^
9	8.33	1.7	125	5.05 ± 0.05	5.88 × 10^4^	6.63 × 10^4^
12	6.67	3.4	125	5.05 ± 0.05	1.22 × 10^5^	6.92 × 10^4^

**Table 2:** Nominal size and modal diameter of droplets from the initial day of manufacturing (D_0_) are compared with the modal diameter of droplets from 33 days (D_33_) after manufacturing. PDI D_0_ is compared with the PDI for D_33_.

Nominal Size (µm)	D_0_ Modal Diameter (µm)	D_33_ Modal Diameter (µm)	D_0_ PDI	D_33_ PDI
1	1.34	1.34	0.068	0.0812
3	2.73	2.73	0.081	0.188
5	4.99	4.91	0.109	0.291
7	6.47	6.47	0.185	0.342
9	8.47	8.47	0.5324	0.5581
12	12.39	12.39	0.0967	0.2224

## Data Availability

The data presented in this study are available from the corresponding author upon reasonable request.

## Supplementary Materials

The following supporting information can be downloaded at: https://www.mdpi.com/article/10.3390/pharmaceutics14112392/s1, Table S1: Average droplet separation for each nominal droplet diameter at the lowest and highest droplet concentrations used.
