# Supplementary material for: Impact of Perfluoropentane Microdroplets Diameter and Concentration on Acoustic Droplet Vaporization Transition Efficiency and Oxygen Scavenging
Source: Pharmaceutics. 2022 Nov 5;14(11):2392. doi: 10.3390/pharmaceutics14112392 (PMC9694497; doi:10.3390/pharmaceutics14112392)
Supplement: Supplementary file 1 [file pharmaceutics-14-02392-s001.zip › pharmaceutics-1948104-supplementary.pdf]

Table S1. Average droplet separation for each nominal droplet diameter at the lowest and highest droplet concentrations used.

Nominal Diameter (μm)	Average distance between droplets (μm) for concentration of 5.1×10^{-5} mL/mL)	Average distance between droplets (μm) for concentration of 6.5×10^{-3} mL/mL)
1	22	4
3	65	13
5	109	22
7	152	30
9	196	39
12	261	52
